# Elevated expression of cholesterol transporter LRP-1 is crucially implicated in the pathobiology of glioblastoma

**DOI:** 10.3389/fneur.2022.1003730

**Published:** 2022-10-04

**Authors:** Shruthi N. R., Minakshi M. Behera, Sanoj Kumar Naik, Sunil Kumar Das, Sooraj Gopan, Amit Ghosh, Rabi Narayan Sahu, Susama Patra, Suvendu Purkait

**Affiliations:** ^1^Department of Pathology and Laboratory Medicine, All India Institute of Medical Sciences, Bhubaneswar, India; ^2^Department of Neurosurgery, All India Institute of Medical Sciences, Bhubaneswar, India; ^3^Department of Physiology, All India Institute of Medical Sciences, Bhubaneswar, India

**Keywords:** Glioblastoma, cholesterol transporter, LRP-1, ABCA-1, receptor associated protein (RAP)

## Abstract

Glioblastoma (GBM) is the most common primary malignant brain tumor with a grave prognosis. The present study evaluated the expression of Cholesterol transporter [importer -Lipoprotein Receptor-related Protein-1 (LRP-1) and exporter -ATP-binding cassette transporters-1 (ABCA-1)] in GBM and their implications in tumor-biology, clinical outcome and therapeutic potentials. The mRNA and protein expression was assessed by qRT-PCR and immunohistochemistry, respectively, in 85 GBMs. For comparison, 25 lower-grade astrocytomas (IDH-mutant, grade-2/3) [LGA] 16 cases of high-grade astrocytomas (IDH-mutant, grade-4) [HGA] were also evaluated. *In-vitro* analysis was performed on U87MG and LN229 glioma cell line. The expression of LRP-1 (mRNA and protein) was significantly higher in GBM than LGA, HGA and normal brain (NB) [p-values 0.007, 0.003 and <0.001 for mRNA; 0.024, <0.001 and <0.001 for immunohistochemistry]. Majority of the GBMs (82.4%) showed strong immunoreactivity for LRP-1, and all tumor cases were positive while the normal brain was negative. LRP-1 immunoreactivity positively correlated with the MIB-1 labeling index (*p*-value-0.013). LRP-1 knockdown *in-vitro* was associated with decreased cell survival, proliferation, migration, invasion, and increased apoptosis. Similar effect was also demonstrated by Receptor Associated Protein (RAP), a LRP-1 inhibitory drug. The silencing of LRP-1 was also associated with decreased cholesterol level. The ABCA-1 expression was higher in GBM than LGA and NB (*p*-value 0.011 and <0.001), however there was no significant association with other parameters. LRP-1 showed a positive correlation with ABCA-1 and associated with decreased expression with LRP-1 knock-down *in-vitro*. The expression of LRP-1 and ABCA-1 didn't correlate with overall survival in GBMs. Hence, LRP-1 is crucial for the tumor cells' survival and aggressive biological behavior which is maintain through the regulation of high intracellular cholesterol import. Its expression is significantly higher in GBMs and also implicated in the regulation of ABCA-1 expression. Considering its immune-positivity only in the neoplastic cell and strong positivity in GBM it may be a useful adjunct to the diagnosis. For the first time, the present study emphasized its role as a potential therapeutic target in the form of RAP which is presently being used in other neurological diseases under clinical trials.

## Introduction

Glioblastoma (GBM) is the most frequently encountered malignant primary brain tumor in adults. Over the last decade, a plethora of genetic/epigenetic research revealed only a limited number of clinically relevant biomarkers, out of which mutation in the Isocitrate dehydrogenase (IDH) gene assumed huge importance. Presently the term GBM is reserved only for IDH wild type grade 4 astrocytic tumors (1). Despite recent advancements in the treatment modalities, the prognosis of GBMs remains dismal (2). In recent years, exploring different aspects of cancer metabolism provides novel insight into cancer biology and paves the path for discovering effective and novel therapeutic targets. One such pathway is cholesterol metabolism. Although the brain is the most cholesterol-enrich organ of the body, its cholesterol pool and metabolisms are separate from the rest as Cholesterol is not permeable through the blood-brain barrier (3, 4). Cholesterol is imported inside the cells predominantly through Lipoprotein receptor-related Protein-1 (LRP-1), a low-density lipoprotein receptor family member. Excess Cholesterol is exported out from the cells largely through ATP-binding cassette transporters-1 (ABCA-1). Apart from cholesterol transport, LRP-1 has a broad spectrum of additional biological functions (5–7).

*In-vitro* studies have shown that Cholesterol is an essential nutrient for the survival and growth of glioma cells. However, the key enzymes involved in the intracellular biosynthesis of Cholesterol were downregulated in GBM cells, indicating that the cells predominantly rely on exogenous Cholesterol to fulfill their metabolic demands (8). In this context, the cholesterol transporters, i.e., LRP-1 and ABCA-1, appeared to be of immense importance in the pathobiology of GBMs.

Altered expression of these cholesterol transporter molecules (LRP-1 and ABCA-1) has been described in ovarian, colorectal, hepatocellular, breast, prostate, and pancreatic carcinomas. The deregulated LRP-1 and ABCA-1 were also correlated with the biological behavior and patient outcome in some of these malignancies (9–15). The limited studies available in glioma assessed the expression of LRP in GBM cell lines or a limited number of tumor samples (16, 17). We could not find any study on ABCA-1 in GBM in published literature. However, in the cell line and the xenograft model, LXR (Liver X receptor) agonist-related GBM cell death was associated with higher ABCA-1 indicating its possible role in the metabolism of GBM cells *in-vitro* (18). We hypothesized that the GBM cells possibly maintain a significant level of intracellular cholesterol by upregulated expression of the importer LRP-1 or downregulation of exporter ABCA-1, or both. Further, this alteration may be crucial for the proliferation and survival of tumor cells.

In the present study, we assessed the implications of these cholesterol transporters in the pathobiology GBMs and compared it with other IDH mutant diffusely infiltrating astrocytic tumors of all grades. We also evaluate the potential of these molecules to be clinically relevant biomarkers and putative therapeutic targets in GBMs.

## Methodology

### Patients and tissue samples

A cross-sectional study was Catasus conducted, including both prospective and retrospective cases. The protocol for the research project has been approved by the Institutional Ethics Committee (T/EMF/PATH/17/03). A total of 86 cases of adult hemispheric GBMs (IDH wild type) were included in the study. For comparison of the expression pattern 16 cases of high-grade astrocytoma, (IDH mutant, grade 4) (HGA), and 25 cases of lower grade astrocytoma (IDH mutant, grade 2/3) (LGA) were also incorporated. Ten cases of normal brain were used as control. The cases with adequate tumor tissue in formalin-fixed paraffin-embedded blocks and snap-frozen tumor tissue stored at −80°C were taken up (2017–2021). We excluded the cases of pediatric cases and diffusely infiltrating midline gliomas.

### Real-time quantitative PCR for mRNA expression

mRNA expression analysis was assessed by real-time quantitative PCR. Fresh tumor tissue specimens were collected in RNA later (Life Technologies) at the time of surgery and stored at −80°C until further use. The details of the procedure is provided in Supplementary Document 1. The expression level (fold change) was calculated by using the 2-^ΔΔ*ct*^ method by taking β-actin as endogenous control (19).

### Immunohistochemical analysis and quantification of immunohistochemistry

Immunohistochemical staining for LRP-1 and ABCA-1 (Table 1) was performed on the manually constructed tissue microarray (5 and 3 mm) (UNITMA, Korea). Scoring for immunohistochemistry was performed by two experienced pathologists (SPu and SPa) blinded to the clinical and histological information. A semiquantitative scoring was ascribed depending on the percentage positivity and the intensity of immunostaining (Table 1). Finally, the immunohistochemical expression was subdivided into low (score ≤ 6) and high (score > 6) expression. There were no discrepancies in the final score between two reviewers (Supplementary Document 1).

**Table 1:** Details of the primers and primary antibody used in the study.

**Primers details**
**Gene**	**Primer sequence (5' – 3')**	**Product size (bp)**	**Annealing Tm (^o^c)**
LRP-1 forward	ACTGCTTGGGGCTGCAAGTC	159	58
LRP-1 reverse	TGCATCAGCCCACACCATGC		
ABCA-1 forward	GGGGCATCATCTACTTCACGCTG	139	60
ABCA-1 reverse	AGGGCAAAGTACTCACAGCCAA		
β-actin forward	AGTATCCTATCGAGCATGGA	102	55
β-actin reverse	CATCTTTTCCCGGTTGATCT		
**Details of primary antibody used**
**Primary antibody**	**Make**	**Dilution**	**Retrieval**
LRP-1	Sigma aldrich	1:400	Tris EDTA pH 9.0
ABCA-1	Novas biologicals	1:200	Tris EDTA pH 9.0
IDH-1	Dianova	1:400	Tris EDTA pH 9.0
Ki67/MIB-1	Dako	Prediluted	Tris EDTA pH 9.0
**Immunohistochemical scoring system for LRP-1 and ABCA-1**
**Variables**		**Score**	**Final score**
Percentage of positively stained tumor cell area	<5%	0	Percentage score X Intensity score Minimum score−0 Maximum score−12 High expression–score ≥6 Low expression–score <6
	5–25%	1	
	>25–50%	2	
	>50–75%	3	
	>75%	4	
Staining intensity (predominant)	No staining	0	
	Weak staining	1	
	Moderate staining	2	
	Strong staining	3	

### Functional study

The human glioma cell line U87MG and LN229 was procured from National Center for Cell Sciences, Pune for *in-vitro* analysis. Cell proliferation, migration, cell cycle and apoptosis were assessed by MTT assay, trans-well-migration assay, and flow cytometry, respectively. Cellular cholesterol levels were measured using the Cholesterol/Cholesterol Ester-Glo™ Assay (Promega, #cat no: J3190) according to manufacturer's protocol (Details in Supplementary Document 1).

### Statistical analysis

Statistical tests were performed using SPSS version 23.0 software (SPSS Inc., Chicago, IL, USA), Microsoft Excel, and Graphpad prism 8 Overall survival (OS) was calculated from t he date of surgery to the date of death due to disease. Patients who were alive at the last follow-up were considered as a censored event in the analysis. To assess the association of various parameters with OS, the Log-rank test was used. In all analyses, two-sided *p* < 0.05 were considered significant.

## Results

### Baseline characteristics

The mean age of the GBM cases was 51.6 years (range 19–72 years). LGA and HGA presented at a relatively younger age group with a mean age of 36.7 years (range 19–52 years) and 40.0 years (range 21–60 years). There was male preponderance (M:F-1.4:1). The frontal lobe being the commonest site for GBM. As described above, all the cases of GBM are IDH wild type while HGA and LGA cases are IDH mutant.

### Expression of LRP-1

#### mRNA expression

**Figure 1:**
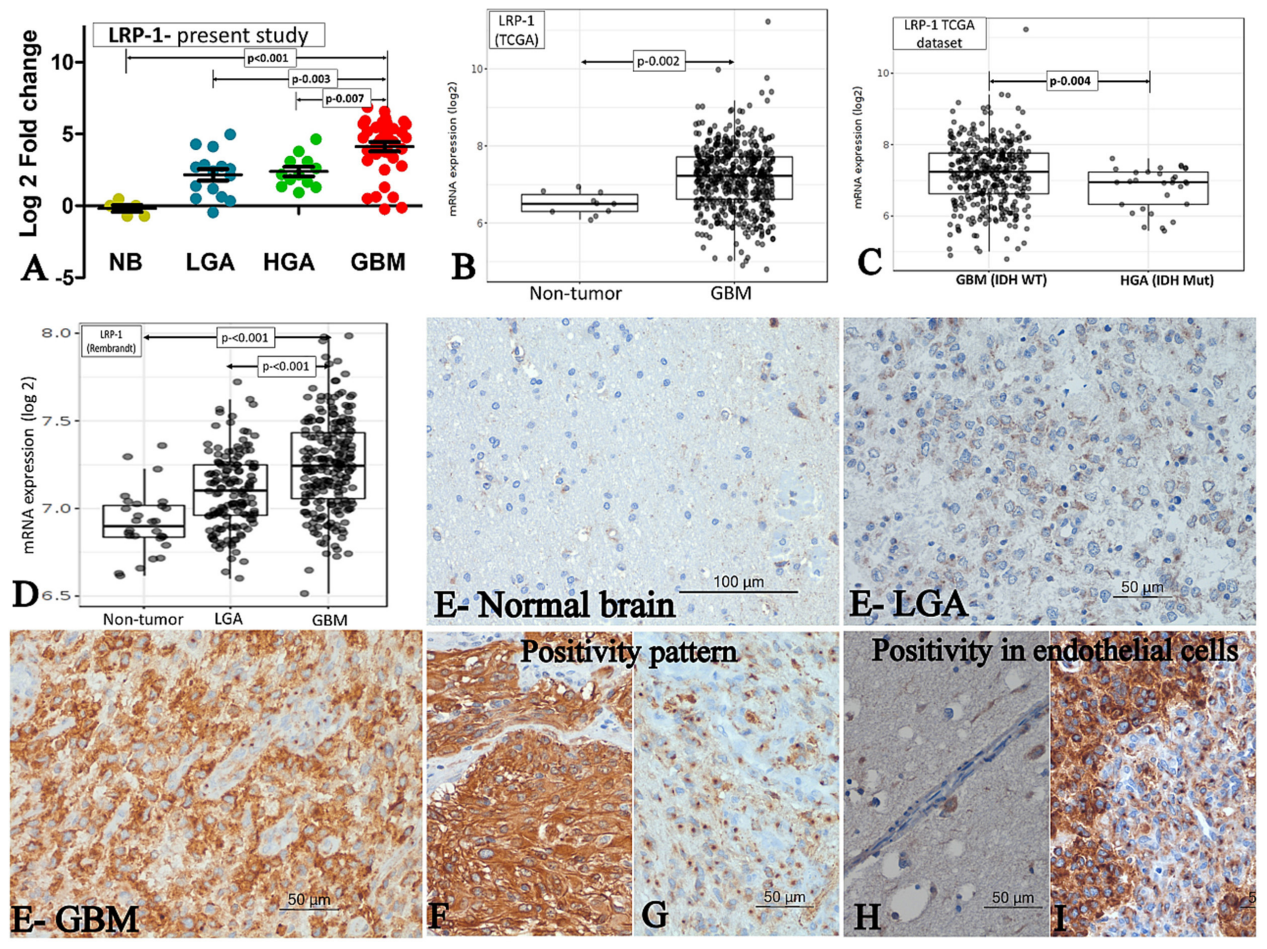
LRP-1 expression. **(A–D)** mRNA expression. The mRNA expression of LRP-1 in cases of GBM and its comparison with IDH mutant lower grade astrocytic tumors (LGA)/ higher grade astrocytic tumors (HGA) in the present study **(A)**, TCGA dataset [normal brain (NB) vs. GBM] **(B)**, TCGA dataset [GBM, IDH wild type vs. Astrocytoma, IDH mutant, grade 4] **(C)** and Rembrandt dataset **(D)**. [Mann-Whitney U test was used to ascertain the *p*-value depicted in the photographs. The parameters compared are denoted by (⇄) sign]. **(E–I)** Immunohistochemistry. LRP-1 immunohistochemistry in normal and tumor samples **(E)**. There was no expression in normal brain (E-normal brain), low expression in LGA (E-LGA) and high expression in GBM (E-GBM). The pattern of expression in tumor cells - Cytoplasmic with membranous **(F)** and Predominant perinuclear dot like **(G)**. LRP-1 immunoreactivity in endothelial cells - negative in normal endothelial cell **(H)**, but positive in proliferating endothelial cells **(I)**.

The mRNA expression was assessed in fresh tissue sample [GBM (*n*- 33), HGA (*n*-12), and LGA (*n*- 15)]. The expression of LRP-1 was significantly higher in GBMs, LGA and HGA compared to the normal brain. Notably, the GBM cases showed significantly higher expression of LRP-1 mRNA than LGA and HGA cases (Table 2, Figure 1A).

**Table 2:** LRP-1 and ABCA-1 mRNA expression and immunohistochemical expression score in GBM (Gr 4), IDH wild type (WT) and comparison with astrocytic tumors (Gr 4), IDH Mut [HGA] and astrocytic tumors (Gr 2/3), IDH Mut [LGA].

		**Histological diagnosis**	**N**	**Median**	**Mean ±SD**	***p*-value**
**LRP-1 Expression**	mRNA (Log2 FC)	Glioblastoma (Gr 4), IDH WT [GBM]	33	4.5	3.9 ± 1.9	**GBM vs. HGA-0.007**
		Astrocytic tumors (Gr 4), IDH Mut [HGA]	12	2.2	2.4 ± 1.1	**GBM vs. LGA-0.003**
		Astrocytic tumors (Gr 2/3), IDH Mut [LGA]	15	2.4	2.1 ± 1.5	HGA vs. LGA – 0.68
	IHC score	Glioblastoma (Gr 4), IDH WT [GBM]	86	9.0	9.2 ± 3.1	**GBM vs. HGA-0.024** **GBM vs. LGA <0.001**
		Astrocytic tumors (Gr 4), IDH Mut [HGA]	16	8.0	6.7 ± 4.5	HGA vs. LGA−0.27
		Astrocytic tumors (Gr 2/3), IDH Mut [LGA]	25	4.0	4.9 ± 3.7	
**ABCA-1 Expression**	mRNA (Log2 FC)	Glioblastoma (Gr 4), IDH WT [GBM]	35	3.1	3.4 ± 2.1	GBM vs. HGA-0.16 **GBM vs. LGA-0.011** HGA vs. LGA−0.25
		Astrocytic tumors (Gr 4), IDH Mut [HGA]	10	1.6	2.3 ± 1.8	
		Astrocytic tumors (Gr 2/3), IDH Mut [LGA]	13	1.2	1.8 ± 1.4	
	IHC score	Glioblastoma (Gr 4), IDH WT [GBM]	85	8.0	7.9 ± 3.4	GBM vs. HGA-0.13
		Astrocytic tumors (Gr 4), IDH Mut [HGA]	16	8.0	6.4 ± 3.0	**GBM vs. LGA-0.008**
		Astrocytic tumors (Gr 2/3), IDH Mut [LGA]	25	6.0	5.8 ± 3.2	HGA vs. LGA−0.40

The values in bold represent highly significant p-values.

#### LRP-1 immunohistochemistry

All cases of normal brain were negative, apart from its focal positivity in the pyramidal neurons. In contrast, all cases of astrocytic tumors showed LRP-1 immunopositivity irrespective of tumor grade. However, the degree of expression was variable. The immunohistochemical expression score was significantly higher in GBMs than LGA and HGA cases (Table 2, Figure 1E). Further, the majority (82.6%, 71/86) of the cases of GBM showed strong expression of LRP-1. In contrast, only 32% (8/25) of cases of LGA and 56.3% (9/16) had strong LRP-1 immunoexpression (*p* < 0.001 and 0.028, respectively).

Interestingly, two distinct patterns of positivity were identified- Weak cytoplasmic positivity with Golgi zone accentuation (Figure 1G) and cytoplasmic with membranous positivity (Figure 1F). We subdivided the cases based on the predominant pattern of immune-expression. Predominant Golgi zone staining was found to be more common in LGA and HGA cases as compared to GBMs [40% (10/25) and 31.2 % (5/16) vs. 17.8% (15/85); *p*-value – 0.028 and 0.30, respectively].

In addition to the tumor cell, LRP-1 positivity was also noted in the proliferating endothelial cell. All the cases of GBMs with endothelial proliferation showed a distinct perinuclear dot-like immunopositivity in the proliferating endothelial cells. In contrast, the endothelial cells in the normal brain were mainly negative or showed a low expression (Figures 1H,I).

#### Correlation between LRP-1 mRNA and protein expression

There was a significant positive correlation between the mRNA fold change and immunohistochemical expression score of LRP-1 (Spearman's correlation coefficient 0.27, *p*-value-0.027).

### Expression of ABCA-1

#### mRNA expression

**Figure 2:**
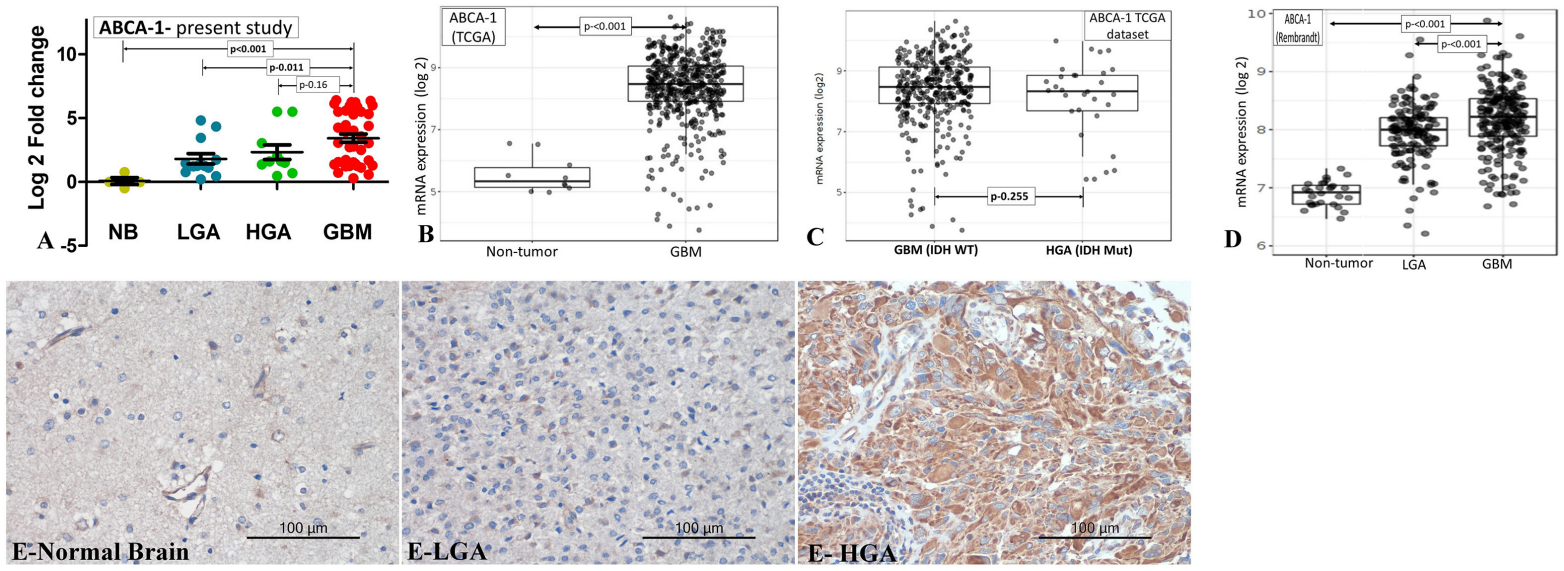
ABCA-1 expression. **(A–D)** mRNA expression. The mRNA expression of ABCA-1 in cases of GBM and its comparison with IDH mutant lower grade astrocytic tumors (LGA)/ higher grade astrocytic tumors (HGA) in the present study **(A)**, TCGA dataset [normal brain vs. GBM] **(B)**, TCGA dataset [GBM, IDH wild type vs. Astrocytoma, IDH mutant, grade 4] **(C)** and Rembrandt dataset **(D)**. [Mann-Whitney U test was used to ascertain the p-value depicted in the photographs. The parameters compared are denoted by (⇄) sign]. ABCA-1 immunohistochemistry in normal and tumor samples **(E)** - very low expression in normal brain (E-normal brain), low expression in LGA (E- LGA) and high expression in GBM (E-GBM).

The mRNA expression was assessed in fresh tissue sample [GBM (n- 35), HGA (n-10), and LGA (n- 13)]. Similar to LRP-1, the expression of ABCA-1 was also significantly higher in cases of all GBM and other diffusely infiltrating astrocytic tumor as compared to the normal brain. The expression in GBM cases was higher as compared to the LGA cases (*p*-value - 0.011). Although, the expression was lower in HGA than GBM, the difference was not statistically significant (Table 2, Figure 2A).

#### ABCA-1 immunohistochemistry

All the cases of the normal brain were positive. However, the positivity was homogenous and of low intensity (immunohistochemistry score: median- 4.0, Mean ± SD-4.8 ± 1.7). The score was higher in GBM as compared to the LGA and normal brain (*p*-value 0.008 and 0.007, respectively). GBM cases also more frequently showed high expression of ABCA-1 (~75%; 64/85) than LGA (44%; 11/25), HGA (50%; 8/16) and normal brain (20%; 2/10) cases (*p*-value – 0.004, 0.06 and 0.001) (Table 2, Figure 2E).

Apart from the tumor cells, endothelial cells (normal and proliferating endothelial cells) also showed positivity for ABCA-1 without a significant difference in the intensity.

#### Correlation between ABCA-1 mRNA and protein expression

There was a significant positive correlation between the mRNA fold change and immunohistochemical expression score of ABCA-1 (Spearman's correlation coefficient 0.23, *p*-value-0.046).

### Correlation of LRP-1 and ABCA-1 expression with proliferative activity in GBM

The LRP-1 protein expression by immunohistochemistry showed a significant positive correlation MIB-1 labeling index (Spearman's correlation coefficient – 0.25, *p*-value 0.020). The cases with high immunoreactivity for LRP-1 showed a significantly higher proliferative activity than those with low LRP-1 expression [Median (mean ±SD) – 20.0 (22.0 ± 8.82) vs. 30.0 (33.8 ± 17.8); *p*-0.013]. There was no significant association of ABCA-1 expression with proliferative activity (Spearman's correlation coefficient – 0.14, *p*-value 0.20).

### Correlation between LRP-1 and ABCA-1 expression

Overall, there was significant positive correlation between LRP-1 and ABCA-1 expression at mRNA (Spearman's correlation coefficient – 0.51, *p*-value 0.001) and protein level (Spearman's correlation coefficient – 0.228, *p*-value 0.003).

### Evaluation of external data-sets for LRP-1 and ABCA-1 expression

We evaluated the Rembrandt and TCGA dataset for the mRNA expression of LRP-1 and ABCA-1. We selected these datasets because of their large size and comprehensive genetics and tumor grading information (19). The LRP-1 and ABCA-1 mRNA expression of the GBM cases was significantly higher than the normal brain/non-tumor tissue and lower-grade astrocytomas (Figures 1, 2B,D). However, these GBMs included both IDH mutant and wild-type cases. Hence, to get a more comprehensive view, we evaluate the cases with respect to IDH status in TCGA. Similar to the present study, the expression of LRP-1 was significantly higher in GBM, IDH wild type as compared to astrocytomas, IDH mutant, grade 4 (*p-*value- 0.004) (Figure 1C). On the other hand, no significant difference was found in the expression of ABCA-1 (Figure 2C).

There was a significant positive correlation between LRP-1 expression and ABCA-1 expression in TCGA and Rembrandt data sets (Correlation coefficient 0.33 and 0.37 respectively; *p* < 0.001 for both).

### *In-vitro* studies

As LRP-1 showed significant differential expression among different tumor subgroups and was associated with proliferative activity, the effect of this molecule was studied in the U87MG and LN229 cell lines. Further, the commercially available inhibitor of LRP-1 was also available, which was previously used clinically in the patient with stroke (20).

#### Effect of LRP-1 gene knock-down on cell viability/survival, proliferative activity and cell migration

**Figure 3:**
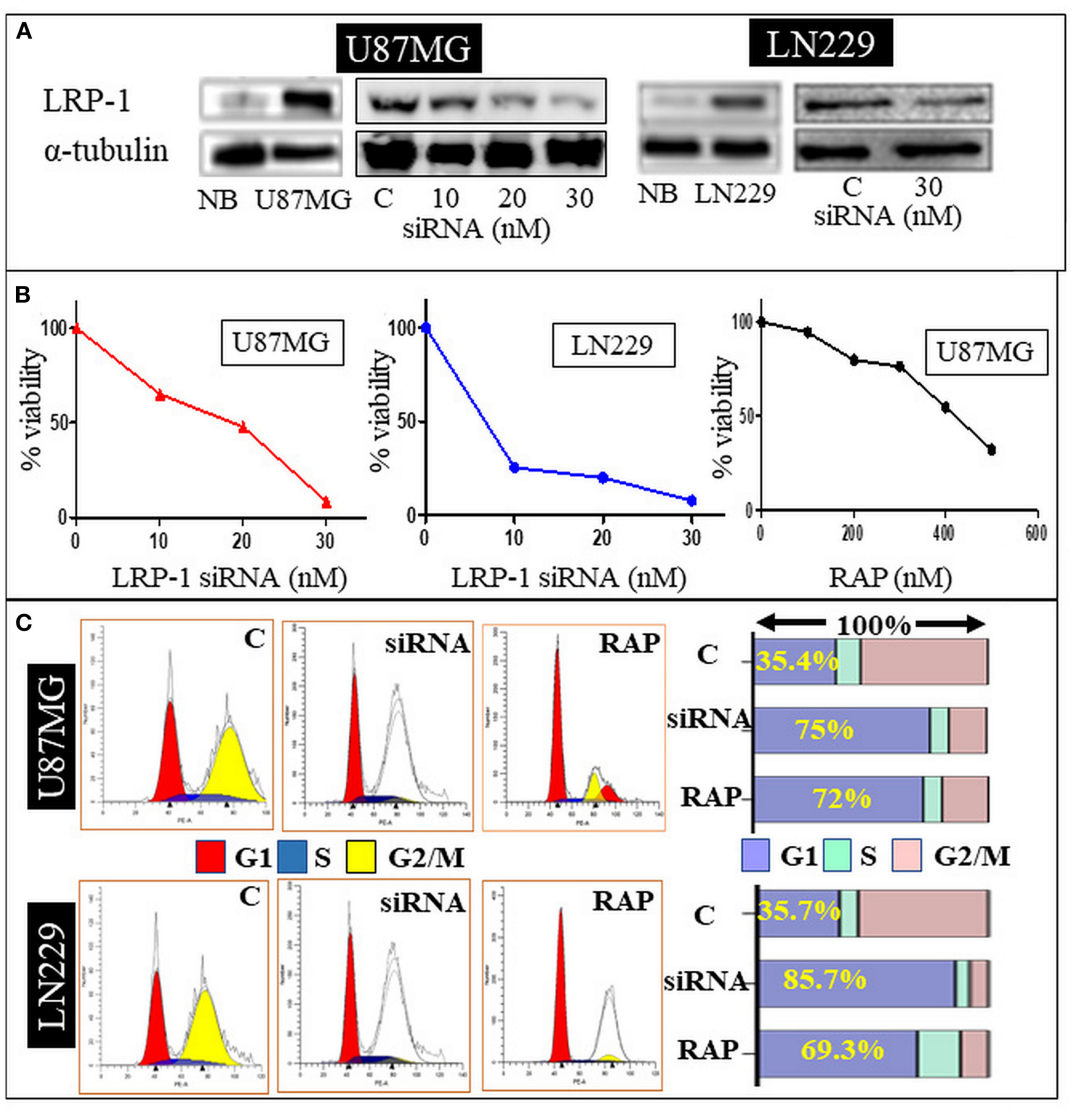
*In-vitro* study. Western blot analysis of siRNA treated U87 MG and LN229 cell line showing a dose dependent decrease in the LRP-1 expression **(A)**. MTT assay showing dose depended decrease in the cell variability and proliferation after LRP-1 knockdown and RAP treatment **(B)**. Cell cycle analysis -Gene knockdown and RAP treatment was associated with increase cell in G1 phase **(C)**.

LRP-1 was significantly upregulated in the U87MG and LN229 glioma cell line at mRNA and protein levels than in the normal brain. The knock-down of LRP-1 by siRNA was confirmed at mRNA and protein levels by qRT-PCR and Western blot analysis (Figure 3A). Both the cell lines showed similar biological behavior after LRP-1 knock-down. MTT assay revealed a significant reduction in the cell viability and proliferation after LRP-1 knock-down, which negatively correlated with the concentration of the siRNA in both the cell lines (Figure 3B). To further evaluate its impact on cell cycle distribution, we assessed the cell cycle after treatment 48 h by PI staining by flow cytometry analysis. LRP-1 knock-down was associated with a significantly increased number of cells in the G1 phase (35.4 vs. 75% in U87MG and 35.7 vs. 85.7% in LN229), indicating possible cell cycle arrest in the G1-S phase (Figure 3C). These data suggest that LRP-1 gene expression is crucial for the viability of the glioma cells and impacts the proliferative activity.

**Figure 4:**
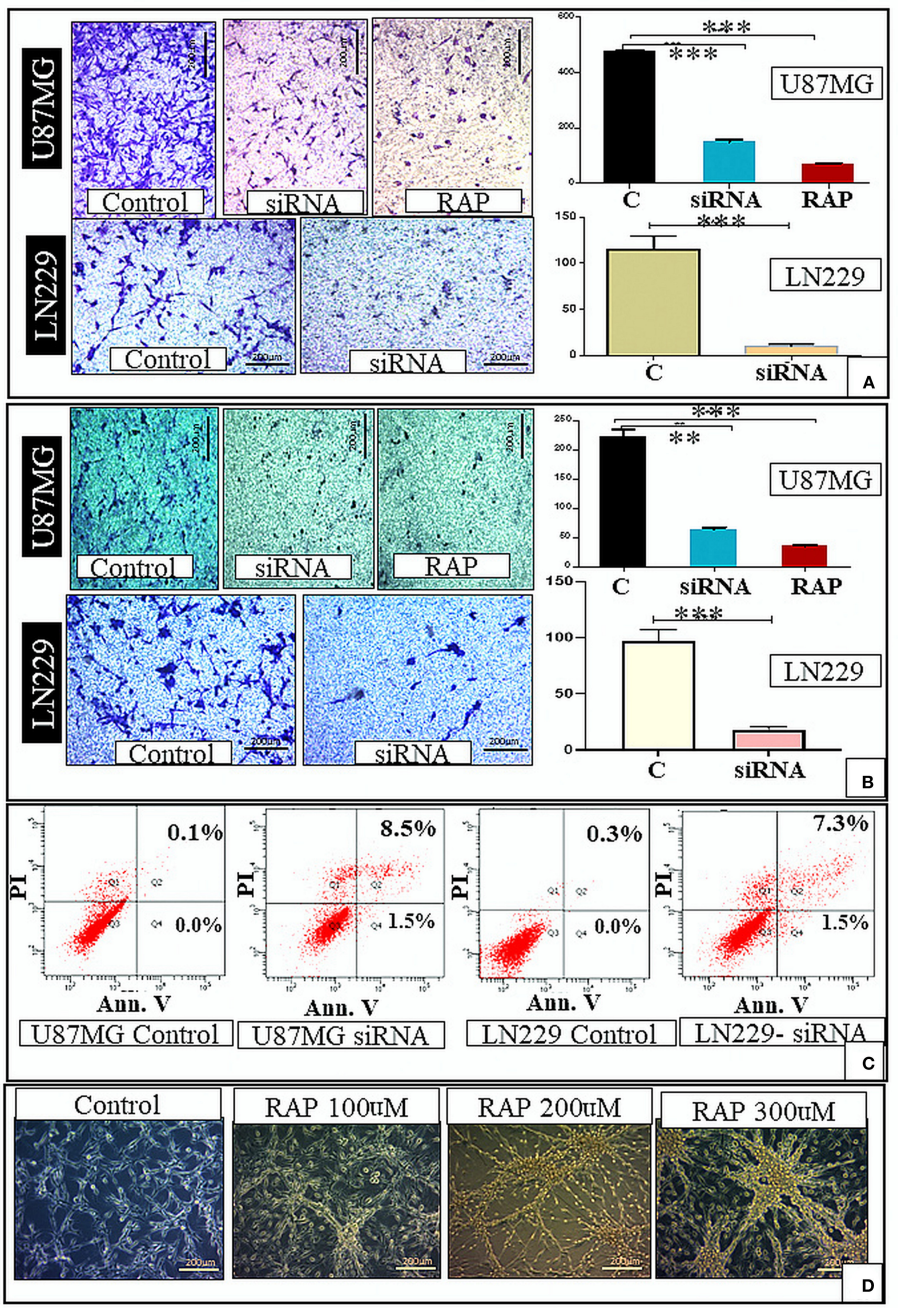
*In-vitro* study. There was decrease in the cell migration after gene knockdown and RAP treatment as compared to the control **(A)**. There was also decrease in the cell invasion after gene knockdown and RAP treatment as compared to the control **(B)**. There was increase in the proportion of apoptotic cells after LRP-1 knockdown in both U87MG and LN229 cell lines (Apoptosis assay) **(C)**. The changes in the U87 cell morphology after treatment with different concentration of RAP is shown in **(D)**. **p < 0.001 and ***p < 0.0001.

Further, the effect of LRP-1 on the cell migration and invasion was assessed by the trans-well-insert. The migratory ability and invasiveness of the tumor cells were also significantly reduced compared to control after LRP-1 knock-down in both the cell lines (Figures 4A,B).

We performed the flow cytometry-based apoptosis assay using Annexin V-PI staining in U87 MG & LN229 cell line. The proportion of apoptotic cells was markedly increased in both the cell line after LRP-1 knock-down. The proportion of cell in early apoptosis (annexin V+ PI-) and late apoptosis (annexin V+ PI+) were increased after LRP-1 knock-down in both the cell lines [control vs. total apoptotic cell population- (0.1 vs. 10%) and (0.3 vs. 8.8%) in U87 and LN229 cell line, respectively] (Figure 4C).

#### Effect of receptor associated protein on cell viability/survival, proliferative activity and cell migration

To determine the effect of RAP on glioma cell line, U87MG and LN229 cells were treated with different concentrations for 48 h. MTT assay revealed the cell viability was gradually decreased in a dose-dependent manner with 50% inhibition occurred at ~ 347.3 μg/mL of drug treatment after 48 h of incubation period as compared to the negative control (Figure 3B). This was statistically significant and depicted that cell viability and proliferative activity were inversely proportional to the drug concentration.

Drug treatment was associated with a significant increase in the cells in the G1 phase, similar to the gene knock-down (Figure 3C). Taken together, RAP targeted LRP-1 suppression has an impact similar to the gene knock-down as described. As RAP attenuated the LRP1 signaling, the effect of RAP on cell migration and invasion were also assessed using trans-well-inserts. The average migrated cells and invasion were significantly reduced in the treated sample than in the control (Figures 4A,B). The concentration (300 nM) and the time taken (48 h) for the assay was not toxic to the cells (Figure 4D), indicating that the inhibition of migration is an intrinsic property of RAP.

#### Effect of LRP-1 gene knock-down on cellular cholesterol level and ABCA-1 expression

**Figure 5:**
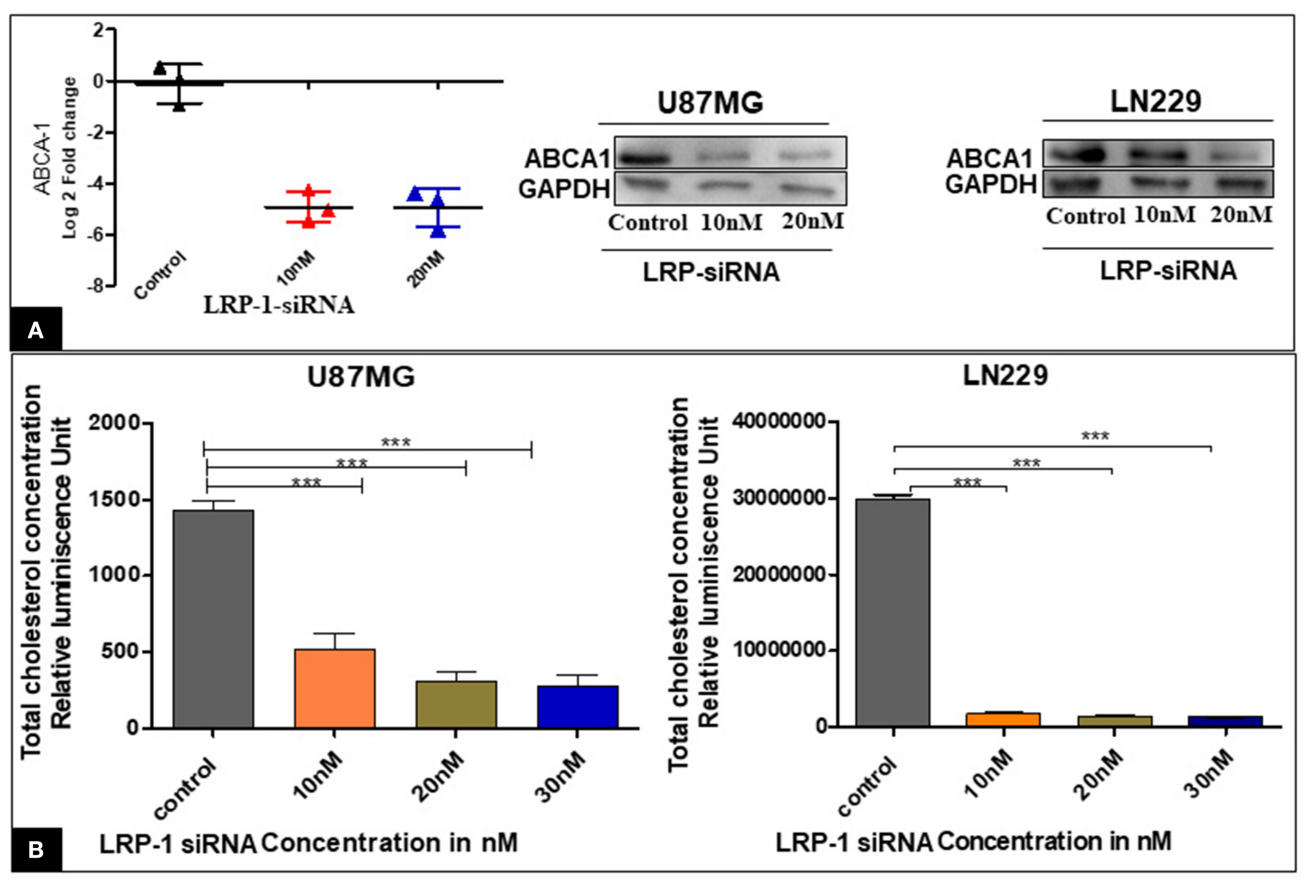
Effect of LRP-1 silencing on ABCA-1 and the cellular cholesterol level. There was a significant decrease in the level of ABCA-1 at mRNA and in protein level after LRP-1 silencing **(A)**. There was also a significant decrease in the cellular cholesterol level after LRP-1 knock- down **(B)**. ***p < 0.0001.

The knock-down of LRP-1 was associated with significantly decreased mRNA and protein level of ABCA-1 (Figure 5A).

The enzymatic Cholesterol/Cholesterol Ester-Glo™ luminometric assay revealed that the proportion of total cholesterol was significantly decreased at different concentrations compared to the control in both the cell line U87MG & LN229 (Figure 5B). Our finding suggested that the inactivation of LRP1 resulted in a significant reduction of cellular cholesterol content.

### Follow-up data

**Figure 6:**
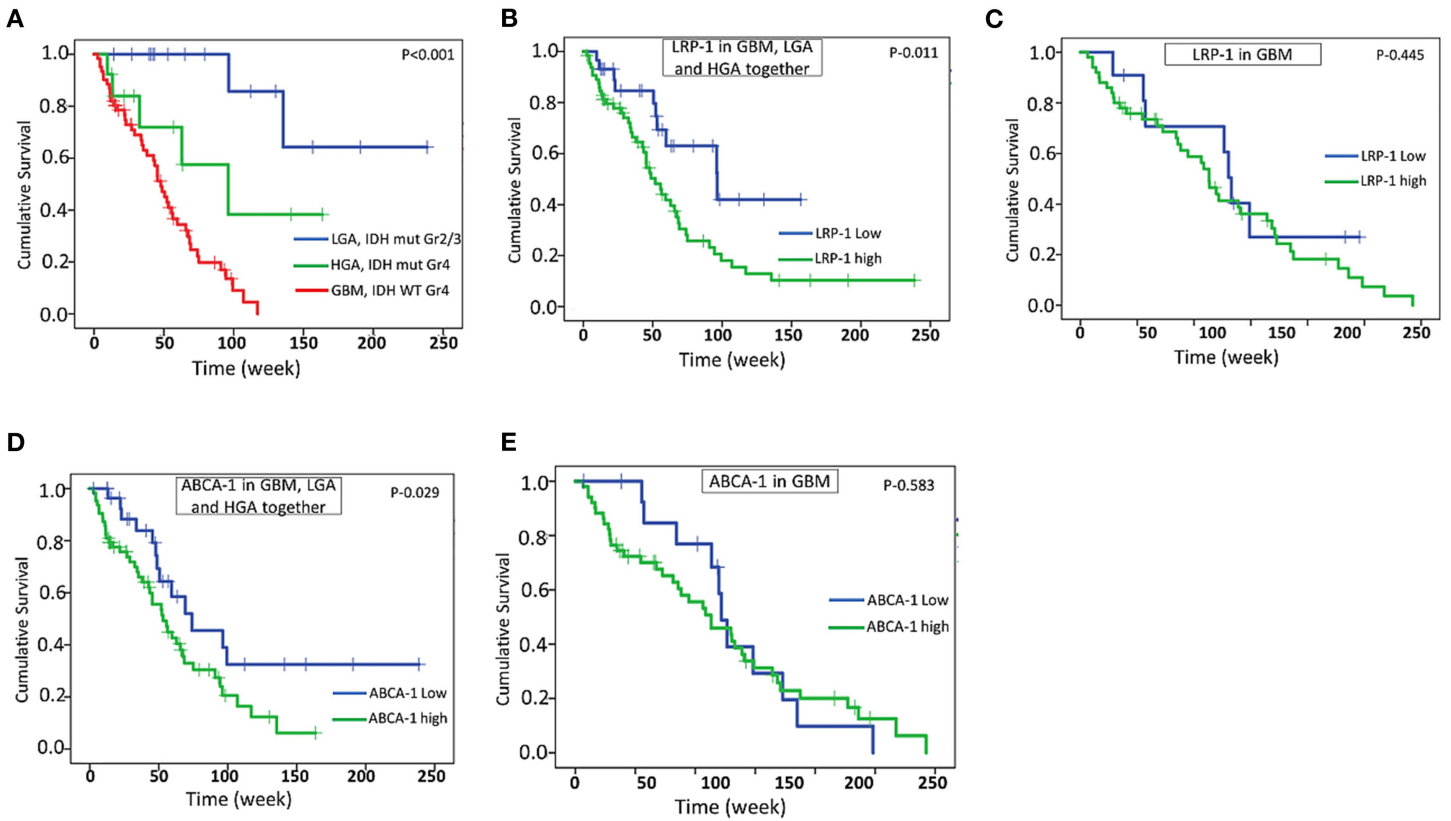
Survival analysis. Kaplan -Meier analysis showing overall survival (OS) in GBM and other IDH mutated astrocytic tumor (HGA and LGA) **(A)**, correlation of LRP-1 expression with OS in all astrocytic tumor and GBM together **(B)**, correlation of LRP-1 expression with OS in GBM separately **(C)**, correlation of ABCA-1 expression with OS in all astrocytic tumor and GBM together **(D)**, correlation of ABCA-1 expression with OS in GBM separately **(E)**. The *p*-values mentioned in the figure are derived from log-rank test.

Follow-up data were available in a total of 61 cases of GBM, 13 cases of HGA, and 16 cases of LGA. The follow-up period ranges from 3 weeks to 238 weeks. All these cases underwent total to near-total surgical resection and had a KPS score of more than 70. As expected, the GBM cases had significantly shorter survival than HGA and LGA (Estimated mean OS – 50.9 weeks vs. 95.9 weeks and 196.2 weeks) (Figure 6A). High protein expression of LRP-1 and ABCA-1 was associated with significantly shorter survival when all the astrocytic tumors, including GBM, LGA, and HGA assessed together (Estimated mean OS for LRP-1 – 70.0 vs. 100.3 weeks; Estimated mean OS for ABCA-1– 62.3 vs. 116.2 weeks) (Figures 6B,D). However, when analyzed in GBM separately, the expression of LRP-1 or ABCA-1 did not significantly affect survival (Estimated mean OS for LRP-1 – 55.8 vs. 49.3 weeks; Estimated mean OS for ABCA-1- – 53.7 vs. 48.2 weeks) (Figures 6C,E). Similarly, in IDH mutated astrocytic tumor cases, there was no significant association of any of these markers with survival.

## Discussion

The present study comprehensively analyzed cholesterol transporter molecules in the GBMs and compared them with other IDH mutated diffusely infiltrating astrocytic tumors considering the present classification scheme. The limited numbers of *in-vitro* studies on various glioma cell lines revealed a considerable degree of variability of LRP-1 mRNA expression with significantly higher levels in SF-539, U-87 MG, and U-343 MG cells lines (16, 17). Two separate studies from the same group of authors qualitatively reported the presence of LRP-1 mRNA in cases of astrocytic tumors by reverse transcriptase PCR. Presence of mRNA was more frequent in GBM cases [87.5% (21/23)−91.7% (11/12)] as compared to anaplastic astrocytoma [50% (4/8)−61.5% (8/13)] and low-grade astrocytoma [42.9% (3/8)−44% (4/9)]. The white matter tissue adjacent to the tumor was negative. Immunohistochemistry done on cases with a positive mRNA band revealed moderate to strong staining in GBMs and weaker staining in LGA (21, 22). Another study on a limited number of cases documented LRP positivity in almost all cases of the astrocytic tumor. No immunoreactivity was identified in the normal brain (23). To the best of our knowledge, no quantitative assessment of mRNA expression is available in the literature in the tumor samples. Further, none of the studies have assessed it in the perspective of IDH status, an important metabolic regulator of diffuse gliomas. The present study highlighted significant upregulation of the cholesterol importer in GBM than the other IDH mutated diffusely infiltrating astrocytic tumors of all grades (grade 2–4). Our *in-silico* analysis of the TCGA and Rembrandt databases also reveals expression patterns similar to the present study. Further, when we subdivided the cases of the TCGA database based on the IDH status, the wild type GBM cases had a significantly higher LRP-1 expression than the IDH mutated grade 4 astrocytic tumor similar to the index study (24). The protein expression as assessed by immunohistochemistry in the present study was also in the line of mRNA expression data with a significant positive correlation between the levels of the two. The recent WHO classification has regarded the IDH mutated GBM as a separate entity and renamed as Astrocytoma, WHO grade 4. When we compared the expression of LRP-1 between the IDH mutated low- (grade 2/3) and high-grade (grade 4) astrocytic tumor, the difference was not statistically significant at protein or mRNA level. Hence, it is possible that IDH may have important role in the expression of LRP-1 in astrocytic tumors. Further, it is known that IDH mutated diffuse gliomas usually have a lower proliferative activity (25). The present study has shown a positive correlation between LRP-1 expression and MIB-1 labeling index. Based on these findings, it may be assumed that IDH mutation status, LRP expression and proliferative activity may be inter-related. However, the present study has not produced any evidence in the favor of the hypothesis.

Considering the predominant immunopositivity of LRP-1 in the tumor tissue only and its high expression in GBM cases, it may be an essential adjunct in routine diagnosis, especially in small biopsies. Further, the present study revealed a distinct topographical variation of LRP-1 expression within the cells. The cases of GBM predominantly showed cytoplasmic and membranous staining, while the LGA cases had a predominant perinuclear Golgi zone immunoreactivity. Although this pattern of immunoreactivity was also highlighted in a previous study, their association with the tumor grade is depicted in the index study (23). The predominant cytoplasmic and surface staining in GBM possibly indicated higher availability of functionally active receptors. Apart from the tumor cells, LRP-1 immunoreactivity has also been reported in the vascular endothelial cells, similar to the index study indicating the possible role of LRP in neovascularization in GBM. Song et al. postulated in a previous study, owing to its positive correlation with urokinase-type plasminogen activator receptor expression, which helps in matrix degradation and endothelial cell migration, a crucial step of neovascularization (26).

One of the novel impacts of the present study is that it established the impact of LRP-1 on the cell biology of GBM and its therapeutic implications. LRP-1 knock-down by siRNA was associated with significantly decreased cell survival, proliferative activity, cell invasion, migratory potential and increased apoptosis of U87MG and LN229 cells. In clinical samples also, high LRP-1 expression was positively correlated with proliferative activity. Previously, Song et al. (26) documented that LRP1 promotes glioblastoma cell migration and invasion by regulating the expression and function of MMP2 and MMP9 (26). LRP-1 tends to exert its proliferative activity through activation of the MAPK pathway which interns activates ERK and inhibits JNK. Together they are responsible for cell proliferation and invasion. Further LRP- 1 has a positive regulatory effect on AKT/ NF-κb pathway (27, 28). A recent study on leukemia has demonstrated the critical role of LRP-1 on the NOTCH pathway. Inhibition of LRP-1 was associated with decreased NOTCH signaling which is one of the important pathways to regulate the proliferative activity of the tumor cells (29). Further, LRP was also found to have a regulatory role on the apoptotic pathway through the caspase 3 enzyme (30). The downstream pathway related to LRP-1 signaling in GBM has not been assessed in the present study.

The RAP-mediated biological effect due to inhibition of LRP-1 is one of the critical findings in the present study. It showed a similar inhibitory effect on the cell biology of GBM like LRP-1 knockdown. RAP is a molecular chaperon which competitively binds to LRP-1 at its ligand binding site and inhibits its action (31–33). This particular drug has already been used in cases of ischemic stroke and has been shown to improve neuro recovery (20). Further, RAP was also found to have a good CNS penetration (32). Hence, RAP may have substantial therapeutic potential in cases of GBMs, especially in patients with elevated LRP-1 expression, which can also be effectively assessed by immunohistochemistry. As LRP-1 expression is very low in the normal brain, these cells will be less affected by the inhibitor therapy. LRP-1 has a critical role in the astroglial differentiation during development and in the clearance of Aβ amyloid in the normal brain (33, 34). siRNA-mediated knock-down of LRP-1 in the mouse primary mixed cortical and hippocampal neuronal cell culture was associated with increased apoptosis due to activation of the caspase 3 (30). On the other hand, in the mouse microglial cell line LRP-1 was associated with inhibition of the microglia. The downregulation of LRP-1 by siRNA and RAP was associated with a proinflammatory micro-environment (35). The action of LRP-1 in the normal brain is complex which is further complicated by their interaction with tumors such as GBM. Hence, the finding of the present study cannot be directly extrapolated and further needed to be evaluated in animal models and clinical trials, which is one of the limitations of the present study.

The literature regarding ABCA-1 expression in GBM is limited. To the best of our knowledge, the present study is the first to describe the ABCA-1 expression status in GBM tumor samples and compare it with LGA. However, no significant difference was found between GBM and HGA. This data was similar to the TCGA and Rembrandt database also showed higher expression in GBM cases (Figures 2B–D) (24). The protein expression also followed a similar trend. Higher expression of ABCA-1 was associated with higher histological grade in ovarian and breast carcinomas (36, 37). In a similar line, its expression correlated with the histological grades of the astrocytic tumors.

The present study also highlighted a decrease in cellular cholesterol level after LRP-1 silencing, indicating its implication as a cholesterol importer in GBM. Interestingly, the present study, for the first time, demonstrated a significant positive correlation of LRP-1 with ABCA-1 in glioma tumor samples as well as in the external data sets. The silencing of LRP-1 was associated with a reduced level of ABCA-1, which was an important finding. The ABCA-1 protein turnover is very rapid and tightly regulated with intercellular cholesterol as an increased amount of unutilized cholesterol contributes to cellular stress (37, 38). Hence, the reduction of intracellular cholesterol due to LRP-1 silencing is possibly associated ted with the down-regulation of ABCA-1 expression.

Both LRP-1 and ABCA-1 high expression was associated with significantly shorter survival when all the astrocytic tumor cases and GBM were assessed together. However, it was not significantly associated with survival when assessed in GBM separately, indicating their limited implication in the prognostication of GBM.

Cholesterol being an integral component of the cell membrane, is essential to sustain the high proliferative activity of GBM. Hence, higher expression of the cholesterol importer LRP-1 as described in the present study is possibly one of the crucial survival machinery of the GBM cell. This is further supported by the positive correlation of LRP-1 protein expression with proliferative activity in the tissue sample and *in-vitro* experiments depicting its association with cell survival, proliferation, migration, and its crucial role in regulating the cellular cholesterol concentration. Further, its expression was relatively lower in all IDH mutated diffusely infiltrating astrocytic tumors (LGA and HGA). Together, the present study provided a novel mechanistic insight into the cell biology of GBMs. The immunohistochemical expression correlated with the mRNA level. On immunohistochemistry, LRP-1 expression was present in all astrocytomas, with the majority of the GBMs showing high expression and was negative in the normal brain. As immunohistochemistry is one of the pathologist-friendly procedures, this marker can be an adjunct for tissue diagnosis. The present study also described the association of LRP-1 with the exporter molecule ABCA-1 which is possibly maintain through the level of intracellular cholesterol.

Most importantly, for the first time, we highlighted that LRP-1 might be an important therapeutic target in GBM. The LRP-1 inhibitor, namely RAP, showed an appreciable anti-tumor effect *in-vitro*. Considering its potential to cross the blood-brain barrier and previous clinical applicability (25), RAP may be a crucial therapeutic adjunct to the present therapeutic regimen of GBM.

## Data availability statement

The original contributions presented in the study are included in the article/Supplementary material, further inquiries can be directed to the corresponding author.

## Ethics statement

The studies involving human participants were reviewed and approved by Institute Ethics Committee All India Institute of Medical Sciences, Bhubaneswar. The patients/participants provided their written informed consent to participate in this study.

## Author contributions

SPu: conception and design. SNR, MB, SN, SG, SPu, and SPa: performed the experiments. SPu, SD, AG, RS, and SPa: contributed reagents, materials, and analysis tools. SPu, SNR, and MB: wrote the paper. All authors contributed to the article and approved the submitted version.

## Funding

This study was funded by Science and Engineering Research Board (SERB), India [Grant No ECR/2017/001646/LS]. SPu has received the grant.

## Conflict of interest

The authors declare that the research was conducted in the absence of any commercial or financial relationships that could be construed as a potential conflict of interest.

## Publisher's note

All claims expressed in this article are solely those of the authors and do not necessarily represent those of their affiliated organizations, or those of the publisher, the editors and the reviewers. Any product that may be evaluated in this article, or claim that may be made by its manufacturer, is not guaranteed or endorsed by the publisher.
