# Supplementary material for: Elevated expression of cholesterol transporter LRP-1 is crucially implicated in the pathobiology of glioblastoma
Source: Front Neurol. 2022 Oct 4;13:1003730. doi: 10.3389/fneur.2022.1003730 (PMC9576951; doi:10.3389/fneur.2022.1003730)
Supplement: Supplementary file 1 [file Data_Sheet_1.docx]

**Supplementary document 1**

1. **Real-time quantitative PCR for mRNA expression**

mRNA expression analysis was assessed by real-time quantitative PCR. Fresh tumor tissue specimens are collected in RNA later (Life Technologies) at the time of surgery and stored at -80 °C until further use. The total RNA was extracted using Trizol reagent (Invitrogen, USA) according to the manufacturer's instructions. DNA contamination was eliminated with RNase-free-DNase set (Life Technologies). The total RNA from samples was quantified using a Nano spectrophotometer (Analytical Technologies). One microgram of RNA was used for cDNA synthesis using Revert Aid First Strand cDNA synthesis kit (#Catalog No: K1622) according to the manufacturer's protocol using gene-specific primers (Imperial life sciences) (Table 1). For qPCR analysis, five ng of cDNA was used, and expression levels were performed in triplicate for each cDNA sample using 2x brilliant III SYBR Green kit in Agilent Aria max (USA) platform. The expression level (fold change) was calculated by using the 2-^∆∆ct^ method by taking β-actin as endogenous control.^[18]^

1. **Immunohistochemical analysis**

Immunohistochemical staining for LRP-1 and ABCA-1 (Table -1) was performed on the manually constructed tissue microarray (5 mm and 3 mm) (UNITMA, Korea). For the construction of tissue microarray, 2-4 cores from each case were taken depending upon the tumor heterogeneity. The immunostains were performed using the standard procedure manually. The details of the primary antibodies are listed in table-1. A positive control (placenta for LRP-1 and normal prostate for ABCA-1) was included during each batch of immunostaining to assess the quality of staining.

1. **Quantification of Immunohistochemistry**

Scoring was performed by two experienced pathologists (SPu and SPa) blinded to the clinical and histological information. A proportion score was calculated in each case as a percentage of the positive tumor area. For this, the slides were scanned under X40 magnification to look for the distribution of the staining and the hot spot (the areas with the highest density of positive nuclei). At least ten high power fields/ a minimum of one thousand tumor cells were counted under X400 magnification starting with the hot spot. The vascular endothelial cells and inflammatory cells were carefully excluded while calculating. A semiquantitative scoring was ascribed depending on the percentage positivity and the intensity of immunostaining (Table 1). Finally, the immunohistochemical expression was subdivided into low (score <6) and high (score ≥ 6) expression. There was no discrepancies in the final score between two reviewers.

1. **Functional study**
   1. **Cell line and Cell culture**

The human glioma cell lines U87MG was procured from National Centre for Cell Sciences, Pune. Cells were routinely maintained in DMEM medium with high glucose and sodium pyruvate (MP Biomedical, USA), supplemented with 10% fetal bovine serum (MP Biomedical, USA) and antibiotics (penicillin 10,000 IU/ml and streptomycin 10 mg/ml and ampicillin 25ug/ml,MP Biomedical) and grown in a sterile humidified chamber at 37 °C, 5% CO_2_ and 95% humidity.

- 1. ***LRP-1 gene knockdown***

For knockdown experiments, siRNA targeting to LRP1 was purchased from Santa Cruz Biotechnology (catlog. Sc 40101). Transfection was done using INTERFERin siRNA Transfection Regent (Ref# 409-10/1ml) according to manufacturer’s guidelines. U87MG cells were subculture 24 hour before transfection to reach 60-70% confluency. All experiments using LRP1 siRNA transfection were done in 48 hours. After 48 hours of trasfection U87MG cells were seeded for migration and MTT assay.

- 1. **Drug treatment**

Receptor associated Protein (RAP) drug was purchased from Sigma-Aldrich (#Cat no. 553506-M) in liquid form at a concentration of 1mg/ml. This is a known anti-LRP-1 agent previously used clinically. Stock concentration was maintained at -80^ο^ C and the cell line was treated with indicated concentration of RAP. Total RNA was isolated from the cell lysate, and gene expression of LRP-1 was analyzed using qRT-PCR, while protein expression was assessed using the immunoblot technique.

- 1. **Cell proliferation assay**

To determine the effect of Receptor associated protein (RAP) on growth pattern/proliferation rate of cancer cells MTT assay was performed by seeding the U87MG cells at a density of 5× 10^3^ cells/well in 96 well plates. The cells were treated with different concentrations of RAP receptor protein (100nM, 200nM, 300nM, 400nM & 500 nM) and incubated for 48 hours. After the incubation period, 10 μl of the MTT labelling reagent was added (final concentration 0.5 mg/ml) to each well and the microplate was incubated for another 3 hours in a humidified atmosphere, at 37 °C, 5% CO_2_ and 95% humidity. Then, 100 μl of the Solubilization buffer was added into each well and the plate was allowed to stand overnight in the incubator in a humidified atmosphere according to manufacturer instruction (Cell Proliferation Kit I (MTT), Cat. no. 11465007001). Total solubilization of the purple formazan crystals was checked and the soluble crystal were quantified using a microplate (ELISA) reader with wavelength of 570nm and reference wavelength of 650 nm. Three independent sets of experiments were performed to evaluate the effect of RAP protein. The graph represents the growth rate. The percentage of survival was calculated by the formula % of Survival = A_570nm_ control/A_570nm_ treated ×100

- 1. **Transwell migrationassay**

Transwell inserts (6 well, 8-µm pore size; Corning, USA) were used to study the migratory abilities of u87MG and LN229 cell line. The cells were pre-treated with LRP-1 siRNA and RAP (300 nM) and seeded at a density of 2.5 × 10^4^ cells /ml in a serum-free medium in the upper well of the Boyden chamber (Corning, USA) and the lower well of the chamber contained media supplemented with 10% FBS. Medium supplemented with 10% serum was used as chemoattractant in the lower chamber. After 48 h, the cells on the upper surface of the filter were wiped out using a cotton swab and the membrane was fixed with 10% formalin and stained with 0.5% crystal violet stain. The membrane was washed 2 times with PBS and the cells that had migrated through the filter to the lower surface were visualized under light microscope. The number of migrated cells in control and RAP treatment were counted in 5 different fields was calculated using ImageJ software and the average value was represented in the graph.

- 1. **Transwell invasion assay:**

For invasion assay, transwell membranes were coated with matrigel (ECM gel from Engelbreth-holm-swarm murine sarcoma, Sigma #cat no: E1270) for 2 h. Then, cells were seeded at density of 5 × 104 cells onto the upper chamber and 500μl medium with 10% FBS was added to the lower chamber. After incubation for 48 h, cells adhering to the upper surface of the membrane were removed with a cotton swab. The invasion or migration cells, which adhered to the lower surface, were fixed with 4% paraformaldehyde and stained with 0.5% crystal violet. Data were obtained from three independent experiments.

- 1. **Cell cycle analysis**

U87MG Cells (3 × 10^5^) were grown in 35mm cell culture plates in Dulbecco’s Modified Eagle Medium supplemented with 10% fetal bovine serum at 37 °C, 5% CO_2_ and 95% humidity for 24 h prior treatment with RAP or siRNA and were allowed to grow for 48 h after treatment. Cells were then harvested and fixed with 70% ethanol and kept at -20°C for 2 hours for fixation. After fixation cells were washed with ice cold 1X PBS thrice and were stained with 1 ml of telford reagent and incubate at 37 °C for 30 minutes. The percentage of cells in each cell cycle phase was measured by flow cytometry on BD FACSCanto II (BD Biosciences) and analysed using Modfit LT v4.1.7 software (Verity Software House, USA).

- 1. **Apoptosis Assay:**

Apoptotic cell death was quantified using the FITC Annexin V apoptosis detection kit II (BD Pharmingen; BD Biosciences). Annexin-V FITC and PI were used to detect apoptotic cell death in both U87mg & LN229 GBM cell line. GBM Cells were collected after 48 h of Si RNA treatment (30nmole) against LRP1along with untreated controls and washed 2 times with PBS. Then, the cells were treated with annexin-V-FITC only, PI only or annexin-V-FITC + PI solution, Unstained and analysed in flow cytometer. FITC Annexin V and PI staining were quantified on 10,000 cells/sample by flow cytometry (FACS Calibur; BD Biosciences; Becton, Dickinson and Company).

- 1. **Cholesterol Measurement:**

Cellular cholesterol levels were measured using the

Amplex

®

Red Cholesterol Assay Kit (Molecular Probes)

according to manufacturer’s protocol. Therefore cells

were treated as indicated for 48 h, detached and either

homogenized right away in a lipid extraction solution

containing chloroform, isopropanol and IGEPAL CA-

630 (7:11:0.1, Sigma) via sonication, or homogenization

was performed on lysosomes isolated as described

previously [45]. After centrifugation (13,000 × g, 10 min)

organic phase was air dried at 50°C for 10 min to remove

chloroform. Remaining organic solvent was removed by

vacuum at 30°C over 30 min. Dried lipids were dissolved

Cellular cholesterol levels were measured using the

Amplex

®

Red Cholesterol Assay Kit (Molecular Probes)

according to manufacturer’s protocol. Therefore cells

were treated as indicated for 48 h, detached and either

homogenized right away in a lipid extraction solution

containing chloroform, isopropanol and

Cellular cholesterol levels were measured using the Cholesterol/Cholesterol Ester-Glo™ Assay (Promega, #cat no: J3190) according to manufacturer’s protocol. After LRP-1 silensing, the culture medium was removed from cells and wash cells twice with 100μl of PBS. Then, add 50μl of Cholesterol Lysis Solution, shake briefly and incubate for 30 minutes at 37°C and add 50μl of Cholesterol Detection Reagent with or without Esterase was added to all wells. Shake the plate for 30–60 seconds by hand or at a low rpm on a plate shaker and after incubating at room temperature for 1 hour, record the luminescence using a plate-reading luminometer. The total and free cholesterol was calculated by comparison of the luminescence (relative light unit, RLU) of samples under the same conditions ( Figure 3).

1. **Statistical analysis**

Statistical tests were performed using SPSS version 23.0 software (SPSS Inc., Chicago, IL, USA), Microsoft Excel, and Graphpad prism 8. To test the association of immunostaining score with other qualitative variables, the Mann– Whitney U test was used. Spearman's correlation coefficients were used for the assessment of correlation between two continuous variables (fold change, immunoreactivity score and MIB-1 labeling index). Overall survival (OS) was calculated from the date of surgery to the date of death due to disease. Patients who were alive at the last follow-up were considered as a censored event in the analysis. To assess the association of various parameters with OS, the Log-rank test was used. In all analyses, two-sided p values <0.05 were considered significant.
